# Remarks on a Review of Dr. Brinton’s Work, Entitled “Ulcer of the Stomach,” by the “American Journal of Medical Sciences,” for July, 1859

**Published:** 1860-11

**Authors:** 


### BOOK AND PAMPHLET NOTICES.

Remarks on a Review of Dr. Brinton's Work, entitled, "Ulcer of the Stomach," by the *American Journal of Medical Sciences*, for July, 1859. By T. Deville, M. D., Associate of King's College, London, late of Ecole Partique, Paris, and Prof. of Anatomy, Lind University.

The reputation of Dr Brinton as an anatomist, physiologist, and pathologist, has been now, for some time, well established in Europe. Few men in medical science have so rapidly risen to eminence ; his investigations have been of a most extended and elaborate nature, and have conferred upon him a distinction of which he may well be proud.

Many of the best articles in the cyclopœdia of anatomy and physiology are from the pen of this highly gifted and talented physician, for example, "Serous and Synoval membranes," "The Eighth pair of Nerves," "Stomach and Intestines," &c. He was made associate of King's College, when the writer of this article became a matriculated student, and afterwards was appointed demonstrator of Anatomy and Medical Tutor. This creditable position gained at an early period of his career, was soon eclipsed by his appointment of lecturer on physiology at St. Thomas' Hospital, and physician to the Royal Free

Hospital, where he has had unbounded opportunities of becoming familiar with diseases, and drawing from them the rich material with which his book abounds.

To him the writer is greatly indebted for the knowledge he possesses in anatomy and physiology, having placed himself under the private tuition of Dr. Brinton, on entering on his college career, and it is but the homage due to this distinguished physician, that the writer now attempts the task of exposing an unfair criticism.

The reviewer contrasts the large materials announced by the author in his preface with the smallness of his results, and not only suggests doubts as to how far the field claimed to have been ranged by the author, has really been examined by him, but specifically questions the likelihood of any physician having met with 200 cases of gastric ulcer in fifteen years of practice, as Dr. Brinton states himself to have done.

So far as it is genuine, the contrast is one the reviewer had a perfect right to make. He is just as much entitled to say that Dr. Brinton has contributed little to our knowledge, as the great bulk of the medical press has had to express or imply the opposite opinion. The *London Medico Chirurgical Review* in four or five successive numbers, devoted a large part of its crowded space to the original researches Dr. Brinton sums up by these lectures. Those scarcely less eminent organs of scientific medicine, the Edinburgh and the Dublin Journals, have paid him high praise expressly for the originality and value of his researches; according to one, original in every line; according to the other, with-drawing successfully the various diseases of the stomach from the domain of conjecture, in which Dr. Abercrombie had permitted them to lie. This favorable verdict seemed confirmed by the translation of different parts of Dr. Brinton's researches into French, German, and Italian reviews. The various weekly journals unanimously came to the same conclusion, and differed from each other only in the seats which they selected for special mention. And as the chief, if not the only fault to these energetic and vivacious organs of medical opinion, is mere liability to a spirit of '*clique*,' it may firmly be surmised that a gentleman whom all agree to be working usefully for the improvement of this branch of medicine, has not been judged on the slender grounds by which a *single critic* is occasionally prejudiced, for or against an author. So that, though the reviewer before us had a right to express his opinion, the profession in America are equally entitled to know *that it is a singular one*, and may fairly be asked to hear it appealed against.

But so far as the reviewer hints or intimates any thing more, or impugns, ever so obliquely, the facts of his author, he stands on a very different footing, and ought, in the name of common truth and honesty, to say either more or less. Dr. Brinton's rising success is not likely to be seriously effected by any carping critic. And though we happen to know that he is one of those who peculiarly values and studies what America is daily adding to medicine and to physiology, and comes of an English family, whose descendants in Philadelphia includes some of our most accomplished and respectable citizens; yet the question is not to be put on grounds even of justice or sympathy to an individual. The profession, which is deeply interested in labors such as his, is just as much interested that labourers of this kind should be appreciated, as that pretenders should be unmasked. The inducements to toils like his are not so many, nor the difficulties and risks so few, as that we can afford to diminish the former, and increase the latter, by what really seems either wonton carelessness or gross injustice.

Come we then to the book itself; and first as regards the observations, the author claims to sum up: twelve hundred records of gastric ulcer, noted during life, and verified after death, form the staple of his statistics. Six hundred of gastric cancers are also quoted; and finally such a series of gastric 'cirrhosis,' dilatation, &c., &c., as imply a range as wide, if not wider, than that which furnished the preceding. Let any one of our readers turn to the latest and best works on this subject; for example—Budd, on the Stomach—or better still, let him walk into the best museum of the largest city he may chance to inhabit, or visit, and we are very much mistaken if a consideration of the statements of the book, or the preparations of this museum would not show that both must be multiplied forty or fifty times over, to give what two of the European reviewers, have well termed the 'vast and elaborate' materials scattered with such unpretending profusion through Dr. Brinton's pages.

We can, however, excuse, if not pardon, the blindness of the Reviewer on one ground: that it is possibly congenital. The most material development, and the wide diffusion of intelligence in Europe, make the concentration and resources of our English scientific brethren a thing very difficult of accomplishment. The rich and numerous libraries and museums at the disposal of a highly educated London physician, have no parallel here. And though we do not wish to detract from the well earned credit Dr. Brinton has gained, of imparting into an obscure and vague department of medicine all the polish and precision of a philosophic inquiry, it is, we are firmly persuaded, neither a fault nor a misfortune that American physic cannot wait fifteen years, and range the literature and museums of half Europe before it writes its book. Suffering from no such metropolitan centralization as English physic is continually growling at, it can afford to receive at second hand these wide and elaborate summaries of disease. The hosts of people whose deaths are utilized in a single book like this, represent a class of diseases to a great extent unknown in this favored country. Want of food and fresh air, destitution of body and depression of mind, so prevalent in most of the great cities of Europe, form a fruitful source of the diseases treated by Dr. Brinton in the work under consideration. And our medical brethren, some of whose works on practical subjects are doing good service in English literature, are no fit objects for condolence in the circumstance that they cannot range a dozzen hospitals, or half-a-dozzen languages, for that slow and daily accretion of materials which inquiries like Dr. Brinton's claim to sum up.

What personal claim he has to belief, is a more delicate question; and one which, if these lines should meet the eyes of himself or his friends, we trust he will excuse us for opening up. But we think credibility of this kind is best decided by evidenee of two kinds: internal and external; the statements themselves, and the results of personal knowledge. As respects the statements, the singular care with which every vagueness or blot is exposed by the author himself, might suffice to vindicate his accuracy. Every word seems to be weighed. His best conjectures, or most seductive theories, are never offered without the opposing facts which threaten, or limit, their value. In short, it is so absurd to imagine any question of his good faith, that we can hardly imagine his reviewer would, on second thought, affect to impugn it. As a matter of analogy, his lectures on Intestinal Obstruction, lately published in the *Lancet*, show him repeating, before the first medical audience in England, the Royal College of Physicians, the results of what he confesses to be a *comparatively imperfect range* of the subject; but yet states to extend over 12,000 *promiscuous necropsies*, and to refer to 600 necropsies of this disease! And in an early volume of the *Pathological Transactions* he casually contributed, with a deprecatory introduction by himself, a table, in which some *sixty cases* of a very rare malady, intra-cranial aneurism, are quoted, each with the specific reference to its original narrator.

The opinion in which his accuracy is held by those who know him personally, is a matter less easy to inquire into: but from all we can learn, he is regarded as unusually scrupulous, and perfectly trustworthy.

The doubt of the *two hundred* cases of gastric ulcer he claims to have had under his own care, is easily disposed of. The writer of this article has reason to know that Dr. Brinton's hospital patients for a long period amounted to the number of 300 weekly. And on turning to the monograph which gives the details of his observations on this disease, it will be found that only 4,000 cases are claimed by him as the yearly number he sees. Remembering the facilities afforded by these hospitals, and the way in which special attention to any particular class of diseases speedily secures to any hospital physician a vast influx of similar cases sent by grateful patients, or professional friends from among the millions of inhabitants in and around London, it seems to us quite certain that, here as elsewhere, there is all the caution of an under-statement, instead of an exaggeration. Indeed, the reports of cases appended to this monograph bear dates which conclusively, though incidently, prove his numbers to be true—fifteen cases selected from the practice of a few months only. On the general value of his results we venture to side with the majority of his reviewers, and against the critic in question. From the first to the last page, every sentence is, as one of the critics has pointed out, carefully weighed; and, we would add, bears the impress of a refreshingly distinct personality. Originality is, indeed, everywhere so marked, that its least obtrusive manifestations go by with scarcely any notice. Our cotemporaries, for instance, generally speak of the introductory chapter on the "Physiology of the Stomach," as a good account which includes the latest discoveries and researches on the subject. And yet there is scarce a part of the book for which the author could more successfully claim originality, and even novelty. The office of the pylorus, the movements of the stomach during digestion, the mechanism of vomiting, and especially the structure of the gastric tubes in reference to the gastric juice, as well as the relation of this secretion to the blood, if not the mechanism of its secretion—all these are treated of, in a manner which is as novel as it is original; and implies no ordinary amount of experimental and inductive skill.

The reviewer's last protest is against the word "cirrhosis" of the stomach; in alluding to the imperfections of which, he, by the way, only follows the author himself, who points them out in a foot-note. As regards "linitis," the term suggested to be preferable by Dr. Brinton, we can only say that it seems as good Greek as most of our nosological terms, and certainly expresses both the nature and seat of the lesion. And since, after all, things must be denoted by some words, we really think that a reviewer who agrees with his author in the necessity for making a new sub-division in the species of any science, ought not to be contented with expressing a vague dislike of the term, which does what he thus confesses to be indispensable; but should show its faults, and suggest a better.

We allow that the style of the book is open to exception. Here again, however, the author in his Preface is beforehand with the critic. It is not intended as a text-book; but addresed to advanced students, and not to the practitioner of thirty years age; and supplemental to the systematic lectures of himself and his colleagues. The book, an *octavo*, and not a duodecimo, as seen by the somewhat prejudiced eyes of the reviewer, corresponds with this purpose; and requires that its reader should either come to its perusal possessed of the average information hitherto known on the subject, or prepared to give his full attention to the terse-pithy sentences and careful qualifications, in which the author deliberately chooses to impart his knowledge. That a book so written should be easy and fluent was impossible, without a latitude of dimensions which would have destroyed their original form; and we would add, that rightly or wrongly, the British public dislikes monographs, and will not read them, or even buy them. So that most men who devote themselves to illustrate and advance any department of medical science, must choose either the general and half-educated reader on the one hand, or the real student on the other.

